# *Cysticercus bovis* in cattle slaughtered in North Egypt: Overestimation by the visual inspection method

**DOI:** 10.14202/vetworld.2021.155-160

**Published:** 2021-01-20

**Authors:** Mona Hassan El-Sayad, Hoda Farag, Hend El-Taweel, Reda Fadly, Nahla Salama, Asmaa Abd Elhameed Ahmed, Naglaa Fathi Abd El-Latif

**Affiliations:** 1 Department of Parasitology, Medical Research Institute, Alexandria University, Egypt; 2 Department of Parasitology, Animal Health Research Institute, Agricultural Research Center, Egypt; 3 Department of Food and Science Technology, Faculty of Science, University of Alexandria, Egypt; 4 Department of Biomedical Informatics and Medical Statistics, Medical Research Institute, University of Alexandria, Egypt

**Keywords:** cattle, *Cysticercus bovis* polymerase chain reaction analysis, *Taenia saginata*, zoonotic

## Abstract

**Background and Aim:**

The World Health Organization and the Food and Agriculture Organization list *Taenia saginata*, a foodborne cestode, as the most widely distributed human tapeworm worldwide. The larval stage of *T. saginata*, *Cysticercus bovis*, causes cysticercosis in bovines and infects humans who eat raw or undercooked beef. The existing detection methods of *C. bovis* in cattle depend on the visual inspection of meat. This study aimed to confirm the identification of *C. bovis* through visual inspection at the slaughterhouses in North Egypt with a molecular diagnosis.

**Materials and Methods:**

A total of 687 locally bred cattle (Baladi), including 428 cows and 259 buffaloes, slaughtered in four slaughterhouses in North Egypt from April 2018 to February 2019 were inspected for *C. bovis* using the traditional meat inspection method. Positive samples were verified through polymerase chain reaction (PCR) amplification and *HDP2* gene sequencing.

**Results:**

Through visual inspection, *C. bovis* was detected in 4.2% and 12.4% of the slaughtered cows and buffaloes, respectively. Molecular analysis confirmed that 1.9% of the animals, all of which were cows, had *C. bovis* infection. DNA sequencing verified the identity of the PCR-amplified product.

**Conclusion:**

The rate of *C. bovis* infection in slaughterhouses detected through meat inspection is overestimated compared with that through PCR. Although meat inspection can be used as a primary screening tool for *C. bovis*, a more specific molecular method is required to achieve an accurate diagnosis.

## Introduction

*Cysticercus bovis* is the larval stage of *Taenia saginat*a. Humans act as its definitive host and cattle as its intermediate host. When an adult worm matures in the small intestine, it releases gravid 3-7 proglottids, each containing 30,000-50,000 eggs per day [1]. Cattle become infected directly by grazing on pasture contaminated with human feces containing *Taenia* eggs or indirectly through sewage sediment or flooding [2]. Humans become infected by eating raw beef containing viable *T. saginata* cysts [3]. An infected bovine carcass could be a source of infection for 8-20 humans [4]. The countries with highly endemic human infection include Central and East Africa countries, such as Ethiopia, Kenya, and Zaire, and the Mediterranean countries, such as Syria, Lebanon, and Yugoslavia [5]. Egypt has an infection rate of 0.6% [6-8].

Cysticercosis in cattle, caused by *C. bovis*, is a leading cause of economic loss in the beef meat industry due to the condemnation of infected meat. The distribution of infection varies with the distribution of taeniasis in humans. Of the 77 million reported cases of bovine taeniasis worldwide, about 40% were from Africa. Ethiopia, with a prevalence reaching 18.49%, has the highest number of cases [9]. In Egypt, the prevalence of *C. bovis* infection is 6.09% in cattle and 9% in buffaloes [6,10].

In slaughterhouses, the primary detection method of bovine cysticercosis is the visual inspection of a carcass’ cut muscles at specific predilection sites, that is, the external and internal masseter pterygoid muscles, heart, tongue, diaphragm, and esophagus. However, this method is inaccurate, as cysticerci can be confused with lesions caused by other organisms, such as *Sarcocystis* and *Actinobacillus*, or other local alterations [11].

The present study aimed to diagnose *C. bovis* through visual inspection at the slaughterhouses in North Egypt and confirm the molecular diagnosis.

## Materials and Methods

### Ethical approval

The study protocol was approved by the Ethical Committee of the Medical Research Institute, University of Alexandria (AU 01219091512). All samples used in the present study were collected postmortem from the discarded infected carcasses unfit for human consumption. No animal was killed for the purpose of this study.

### Study period, location and sampling

This study was conducted from April 2018 until the end of February 2019 in four abattoirs (Kom Hamada, Damanhur, and Kafr El Dawar in El-Beheira Province, and Tanta in El-Gharbia Province) in North Egypt. A total of 687 locally bred cattle, 428 cows and 259 buffaloes aged below 6, underwent postmortem examination and detailed visual inspection for *C. bovis* at six deep cuts in the relevant organs.

### Visual examination and cyst classification

The suspected cases of *C. bovis* were classified as viable or degenerating following macroscopic examination and finger palpation. Translucent, fluid-filled cysts were considered viable, whereas empty cysts and those with cheesy or solid contents were considered degenerating or non-viable [12,13].

### Molecular analysis

DNA was extracted from all the detected cysts using the DNeasy Blood and Tissue Mini Kit (Qiagen, Hilden, Germany) according to the manufacturer’s protocol. Conventional polymerase chain reaction (PCR) was performed to detect *C. bovis* *HDP2* gene using primers described by Fahmy *et al*. [6].

DNA amplification was performed in 25 μL reactions containing 3 μL of template DNA, 10 pmol of a forward primer (PTs7S35F1; 5′- CAGTGGCATAGCAGAGGAGGAA-3′) and 10 pmol of a reverse primer (PTs7S35R1; 5′-GGACGAAGAATGGAGTTGAAGGT-3′), 12.5 μL of Taq PCR Master Mix (MyTaq™ HS Red Mix, Bioline, UK), and 7.5 μL of nuclease-free water. The reactions underwent denaturation at 95°C for 3 min, followed by 35 cycles of denaturation at 95°C for 15 s, annealing at 55°C for 15 s, and extension at 72°C for 20 s and a final extension step at 72°C for 10 min. To detect the expected 599 bp band of amplified DNA, the products were separated on 1.5% agarose gel and stained with ethidium bromide [14]. Nuclease-free water was used as a negative control for every PCR run.

### DNA sequencing

Two representative positive PCR samples were chosen for DNA sequencing using the forward primer (PTs7S35F1) with the ABI PRISM^®^ 3100 Genetic Analyzer according to the manufacturer’s protocol (Micron-Corp., Korea). The resultant sequences were analyzed using the BLAST program at NCBI [15].

### Statistical analysis

The data were analyzed using the Statistical Package for the Social Sciences software version 20 (Chicago, IL, USA). The quantitative data were expressed as numbers and percentages. The difference between the categorical variables was tested using Pearson’s Chi-squared test and Fisher’s exact p-value (FEp) for 2×2 tables if there was at least one cell with an expected frequency of <5. The Monte Carlo significance test was employed for r×c tables if more than 25% of cells have an expected cell count of <5. p-value of less than 0.05 was considered significant.

## Results

### Detection by visual examination

Postmortem visual examination revealed that out of the 687 cattle (428 cows and 259 buffaloes), 7.3%, or 50 animals of 18 cows and 32 buffaloes, were likely infected with *C. bovis* cysts. The infection rates were 4.2% and 12.4% in cows and buffaloes, respectively. No statistically significant difference was observed in the cows’ infection rates (MCp=0.720) between the abattoirs. However, there was a statistically significant correlation between the abattoirs and the occurrence of infection in buffaloes; in Kom Hamada, the infection rate was 7.3%, whereas in Tanta, it was 41% (FEp<0.001) (Table-1).

**Table-1:** Prevalence of suspected *Cysticercus bovis* in 687 slaughtered cattle in four official abattoirs in Egypt.

Abattoirs	Cows			Buffaloes		
	
	Number of examined	Infected		Number of examined	Infected	

		n	%	n	%	
Kom Hamada	6	1	16.7	220	16	7.3
Kafr El-Dawar	15	1	6.7	0	-	-
Damanhur	383	15	3.9	0	-	-
Tanta	24	1	4.2	39	16	41
Total	428	18	4.2	259	32	12.4
p-value	MCP=0.720			FEP<0.001*		

MCp=Monte Carlo significance, FEp=Fisher’s exact significance,

* Statistically significant at p≤0.05

The infection rate in cattle by age was examined (Table-2). Compared with 4.8% of cows aged above 2 years, 3.2% of cows below 2 years were infected. Compared with 16.1% of buffaloes aged above 2 years, 7.3% of those below 2 years were infected, thus exhibiting a statistically significant difference (p=0.033).

**Table-2:** Association between suspected *Cysticercus bovis* infection and age of cattle in cows and buffaloes.

Age in years	Cows		Buffaloes	
	
	Infected	Not infected	Infected	Not infected
<2	5 (3.2)	151 (96.8)	8 (7.3)	102 (92.7)
≥2	13 (4.8)	259 (85.2)	24 (16.1)	125 (83.9)
χ^2^(p-value)	0.61 (0.43)		4.56 (0.033)*1	

χ^2^=Chi-square test, p=p-value for comparing between the studied groups,

* statistically significant at p≤0.05

All the examined cows were male, and all the buffaloes except one were female; the infection rates were 12% and 100% for cows and buffaloes, respectively. Among the buffaloes, the infection rates by sex were not statistically significant, but in cows, these rates could not be assessed (Table-3).

**Table-3:** Association between suspected *Cysticercus bovis* infection and sex of cattle in cows and buffaloes.

Gender	Cows		Buffaloes	
	
	Infected	Not infected	Infected	Not infected
Males	18 (4.2)	410 (95.8)	1 (100)	0 (0)
Females	0 (0)	0 (0)	31 (12)	227 (88)
Total	18	410	32	227
p-value	--------		FEP=0.135	

FEp=Fisher’s exact significance, p=p-value for comparing between the studied groups, *statistically significant at p≤0.05

**Figure-1:**
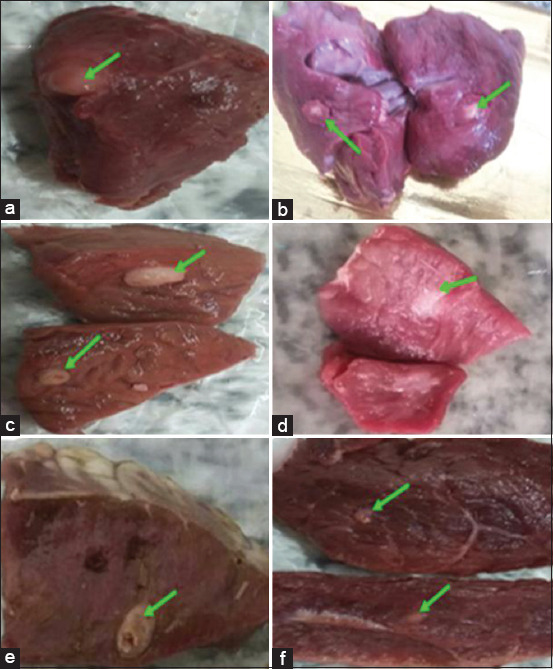
(a-f) Suspected viable *Cysticercus bovis* cysts collected from infected slaughtered cattle in Damanhur and Kafr El-Dawar abattoirs.

**Figure-2:**
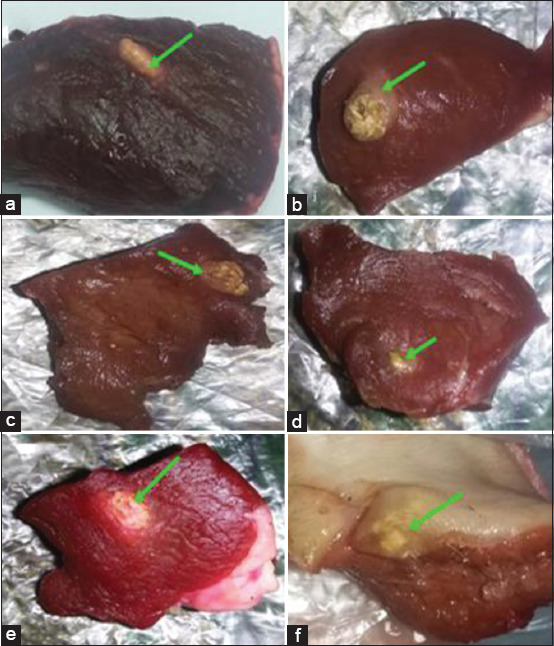
(a-f) Suspected degenerating (non-viable) Cysticercus bovis cysts collected from infected slaughtered cattle in Damanhur abattoirs.

The anatomical distribution and viability of the visually diagnosed cysts were also analyzed (Table-4). Of the 50 collected cysts, 2 were recovered from masseters, 18 from tongues, 15 from heart tissues, and 15 from the esophagus. Of the 50 cysts, 13 cysts (26%) were viable, whereas 37 (74%) were non-viable. The morphological appearance of viable and non-viable cysts was included (Figures-1 and 2).

**Table-4:** Anatomical distribution and viability of suspected *Cysticercus bovis* in inspected organs of slaughtered cattle.

Organs	Collected cysts		Examination			

			Viable cysts		Non-viable cysts	
		
	n	%	n	%	n	%
Masseter	2	4	1	50	1	50
Tongue	18	36	3	16.7	15	83.3
Heart	15	30	7	46.7	8	53.3
Esophagus	15	30	2	13.3	13	86.7
Total	50	100	13	26	37	74

The distribution of infection in various seasons was also analyzed (Table-5). A statistically significant difference was observed in the infection rates between seasons, that is, higher in summer at 46% and autumn at 40%, than spring at 10% and winter at 4% (p<0.001).

**Table-5:** Distribution of the suspected infected cattle by season.

Seasons	Number of examined	Infected	

		n	%
Spring	95	5	10
Summer	485	23	46
Autumn	83	20	40
Winter	24	2	4
Total	687	50	100
χ^2^(p-value)		40.022 (<0.001)*	

χ^2^=Chi-square test, p=p-value for comparing between the studied groups,

* statistically significant at p≤0.05

### Molecular analysis

**Figure-3:**
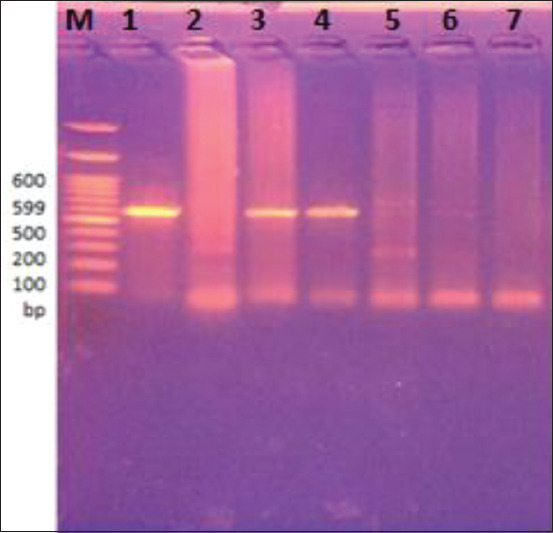
Electrophoretic separation of conventional polymerase chain reaction product of DNA amplified amplicon of *HDP2* gene isolates of suspected *Cysticercus bovis* cysts resolved by 1.5% agarose gel electrophoresis and visualized by ethidium bromide staining at 599 bp; where M is the molecular weight marker (100 bp ladder). Lanes 1, 3, and 4 show positive clear bands of size 599 bp, corresponding to the expected *C. bovis*. Lanes 2, 5, and 6 illustrate negative results. Lane 7 represents negative control (nuclease-free water).

Out of the 50 suspected cysts collected from cows, 13 (26%) were confirmed through PCR (Figure-3). Thus, the actual infection rate of *C. bovis* in the examined cows was 3% or 13 out of 428. However, none of the cysts recovered from buffaloes was confirmed through PCR. Contrarily, the infection rate was significantly higher in buffaloes at 12.4% than cows at 4.2% by visual examination (Table-6).

**Table-6:** Infection rates among slaughtered cattle by animal type, age, and sex of animals using visual examination and conventional PCR.

Infection	Cattle positive by visual examination			Cattle positive by PCR		
	
	n	%	χ^2^ (p-value)	n	%	χ^2^ (p-value)
Overall prevalence	50	7.3		13	1.9	
Infection by animal type						
Cows (n=428)	18	4.2	4.5 (0.03)*	13	3	-------
Buffaloes (n=259)	32	12.4		0	0	
Infection by age						
<2 (n=266)	31	4.9	3.1 (0.07)*	5	1.9	0.07 (0.7)
≥2 (n=421)	37	8.8		8	1.9	
Infection by sex						
Males (n=429)	19	4.4	12.6 (<0.001)*	13	3	-------
Females (n=258)	31	12.01		0	0	

χ^2^=Chi-square test, p=p-value for comparing between the different categories,

* statistically significant at p≤0.05. PCR=Polymerase chain reaction

According to PCR analysis, the proportions of viable and non-viable cysts were comparable at 53.8% and 48.2%, respectively. According to the analysis by anatomical distribution, 84.6% of PCR confirmed that *C. bovis* cysts were found in the heart (Table-7).

**Table-7:** Distribution and viability of *Cysticercus bovis* in inspected organs of slaughtered cows as diagnosed by conventional PCR.

Organs	PCR-positive cysts		Viability•			
	
	n	%	Viable		Non-viable	
	
			n	%	n	%
Masseter	1	7.7	1	100	0	0
Tongue	1	7.7	0	0	1	100
Heart	11	84.6	6	54.5	5	45.5
Total	13	100	7	53.8	6	46.2

• Viability was tested by macroscopic examination. PCR=Polymerase chain reaction

With regard to age, no statistically significant difference was observed in the infection rates between cattle aged below and above 2 years by visual examination (p=0.07) or PCR analysis (p=0.7). With regard to sex, a statistically significant difference was observed in the infection rates between male and female cattle by visual examination (p<0.001). However, all the infected cattle confirmed through PCR were male.

### Sequencing

Two randomly selected samples were sequenced to confirm the identity of the PCR product. The two sequences were submitted to GenBank with accession numbers MN862281 and MN862282. Through sequencing analysis, the best match in the sequence of *T. saginata* intragenic spacer (IGS) isolate 5 (FM212967.1) and isolate 2 (FM212965.1) (https://blast.ncbi.nlm.nih.gov/Blast.cgi) was identified.

## Discussion

In livestock production, bovine cysticercosis is a significant problem. It causes severe economic loss in the meat industry due to meat condemnation or carcass downgrading [16].

The current detection method of cysticercosis is based on the postmortem inspection of carcasses. Accordingly, this method has been employed as the first line of diagnosis. The overall infection rate in the examined cattle was 7.3%, higher than that reported in Egypt by Fahmy *et al*. [6], which was 6.09%, and comparable to that detected by Dyab[8], which was 7.5%, using the same method. However, the infection was noticeably less intense, and buffaloes were infected at relatively lower rates than cows.

The infection rate determined by the present results was higher than those recorded in Iran at 0.25% [17], Nigeria at 3% [18], and Ethiopia at 3.6% [19] but lower than those recorded in Ethiopia at 22.9-26% [20,21]. The variation in the infection rates may be due to epidemiological factors, such as climate, different sanitation measures, number of the collected samples, and application of control measures and programs [18].

The analysis of the age distribution of cystic lesions revealed that the infection rate was significantly high among older buffaloes and might represent cumulative exposure to the parasite. The present results were consistent with those recorded by Dorny *et al*. [22]. However, Oryan *et al*. [23] reported no variation in the infection rate in cattle by age. However, the infection did not significantly vary by sex among buffaloes and could not be assessed in cows.

Out of the 50 cysts diagnosed visually, there were fewer viable cysts than degenerated ones; this observation was in accordance with Basem *et al*. [7]. The analysis of the anatomical distribution of the suspected *C. bovis* cysts revealed that the cysts were almost equally distributed in the tongue, heart, and esophagus; however, few were found in the masseter. These findings were inconsistent with the previous findings [7,9]. The variations in the number of *C. bovi*s found in predilection sites may be attributed to the experience of the meat inspectors in identifying infected animals and accurately diagnosing the cysts. The variation in the predilection sites may also depend on the use of animals in different agricultural activities that may influence blood kinetics that affects the distribution of oncospheres [8].

Infection was found to be highest during summer and autumn. Seasons may affect the eggs’ development, survival, and access to the grazing cattle; thus, temperature and humidity may play a role in the epidemiology of cysticercosis. However, the present finding is inconsistent with those of Usip *et al*. [18].

In general, the diagnosis of cysticercosis through meat inspection is reportedly ineffective, particularly in countries with low prevalence of such infection. False identification of morphologically similar lesions caused by other tissue parasiteshas been reported [11]. Therefore, a detailed examination with more incisions in the heart is recommended [16].

In the current work, conventional PCR was performed as a second-line diagnosis, which revealed that only 13 out of the 50 collected cysts were caused by *C. bovis*. The actual infection rate was lower (1.9%) than that reported through visual inspection. None of the buffaloes were molecularly diagnosed to be infected with cysticercosis. The cysticercosis-negative samples were later tested and found to be positive for *Sarcocystis* spp. cysts (data not shown; manuscript in progress). PCR could detect viable and non-viable *C. bovis* cysts. Contrary to the visual inspection method, the heart was identified as the primary predilection site for *C. bovis*. These findings were consistent with those reported by Regassa *et al*. [24]. Although the PCR-negative cysts could not be identified, molecular diagnosis could be employed to avoid the considerable and unnecessary loss of meat with a suspected *C. bovis* infection.

## Conclusion

Our study has demonstrated that meat inspection has resulted in an overestimation of cysticercosis in the locally bred cattle. Although the visual inspection is critical for the examination of many slaughtered animals, more sensitive and specific methods are required to confirm the diagnosis of cysticercosis.

## Authors’ Contributions

MHE conceived and designed the work and conducted the field work together with HF, HE, RF, NFA, and NS. MHE, NFA, and HF supervised the study. NFA, RF, AAA, and NS performed laboratory work, statistical analysis, interpreted the data, and drafted the manuscript. MHE, NFA, and HE edited and reviewed the manuscript. All authors read and approved the final manuscript.
